# Assessing the impact of acute severe hypertension in the emergency department: A prospective cohort study in Karachi, Pakistan

**DOI:** 10.1371/journal.pgph.0003948

**Published:** 2024-12-04

**Authors:** Junaid A. Razzak, Noman Ali, Uzma Khan, Madiha Ismail, Badar Afzal Khan, Ahmed Raheem, Priyanka Agrawal, Junaid Bhatti

**Affiliations:** 1 Department of Emergency Medicine, Weill Cornell Medicine New York, New York, NY, United State of America; 2 Department of Emergency Medicine, Aga Khan University, Karachi, Pakistan; 3 Department of Emergency Medicine, Johns Hopkins University, Baltimore, MD, United State of America; 4 Manulife Canada, Toronto, Ontario, Canada; Institute for Epidemiology, Biostatistics and Prevention, University of Zurich, SWITZERLAND

## Abstract

Acute Severe Hypertension (ASH), presenting as a Hypertensive Emergency (HE) or Hypertensive Urgency (HU), is a frequent reason for emergency department (ED) admissions. This study sought to assess the prevalence of ASH among adult ED patients in Pakistan and investigate all-cause mortality and hospitalization rates over six months. We conducted a prospective single-center cohort study in Karachi, Pakistan, from June 3, 2019, to September 22, 2020. We enrolled all adult male and non-pregnant female patients presenting to the emergency department with a systolic blood pressure of ≥180 mm Hg or diastolic blood pressure of ≥120 mm Hg. Telephonic follow-ups were conducted at one, three-, and six months post-discharge from the hospital. The Cox Regression Model was used to identify the risk factors for mortality. **O**f 49,431 ED visits during the study period, 1,525 (3.1%) met the inclusion criteria, and 1,161 (76.2%) were enrolled. A total of 356 patients (30.6%) were diagnosed with HE, and 805 (69.2%) with HU. Among follow-up patients, 14.6% with HE and 4.7% with HU experienced mortality within six months. Notably, the risk of mortality was higher in patients aged >65 years (aRR = 1.90, 95% CI = 1.20 to 3.02) and those suffering from stroke (aRR = 2.09, 95% CI = 1.21 to 3.61) or acute kidney injury (aRR = 1.82, 95% CI = 1.09 to 3.04). Conversely, regular blood pressure monitoring (aRR = 0.08, 95% CI = 0.03-0.19) and adherence to antihypertensive medications (aRR = 0.23, 95% CI = 0.09-0.56) significantly lowered the risk HE resulted in heightened mortality at six months, while HU, traditionally deemed benign, also led to substantial morbidity and mortality. This underscores the ED visit for ASH as a crucial opportunity for preventing short-term and longer-term health complications.

## Introduction

Hypertension is the leading preventable risk factor for cardiovascular disease and all-cause mortality worldwide, affecting one-third of the global population, especially in low and middle-income countries (LMICs) [1–3]. Effective blood pressure control is a major public health objective aligned with the global Sustainable Development Goals [4].

Despite effective and affordable treatment, nearly a billion people globally have uncontrolled hypertension [5], and approximately 2% of these patients develop severe hypertension and represent up to 5% of emergency department (ED) admissions in some regions [6]. Data from high-income countries show an increasing trend for hypertension-related emergency visits and admissions [7, 8].

The diagnosis of Acute Severe Hypertension (ASH) or Hypertensive Crisis is assigned to individuals with systolic blood pressure > 180 mmHg or diastolic blood pressure > 120 mmHg. ASH is further stratified into Hypertensive Emergency (HE) or Hypertensive Urgency (HU), based upon the presence or absence of damage to vital end-organ such as the brain, retina, heart, blood vessels, or kidneys. While immediate and careful treatment of HE significantly improves outcomes, “urgency” in HU is increasingly scrutinized due to the absence of demonstrable benefits and the potential risks associated with immediate intervention for this "Markedly elevated BP" in acute settings [9, 10].

While Hypertensive Emergencies (HEs) are known to cause high in-hospital mortality rates, prospective data on the post-acute care outcomes of patients with HE and HU are notably absent, especially in LMICs [11–14]. Existing studies with follow-up data are predominantly derived from high-income countries, relying on retrospective and secondary data sources. Notably, these studies have limited follow-up periods, and only a few address patients with HU [15–23]. Our investigation aimed to address this gap in knowledge by assessing the prevalence of Acute Severe Hypertension (ASH) among adult patients presenting to the ED in Pakistan and quantifying all-cause mortality and hospitalizations over six months following their initial visits to the emergency department.

## Methods

### Study design and study population

This prospective observational study was conducted at the ED of Aga Khan University Hospital (AKUH) in Karachi, Pakistan, from June 3, 2019, to September 22, 2020, with an exclusion period from March 16, 2020, to July 28, 2020, due to the COVID-19 pandemic.

### Setting

AKUH, a 710-bed private, fee-for-service teaching, and major referral hospital, meets the diverse healthcare needs of Karachi, a city with a population exceeding 20 million. AKUH’s ED annually attends to around 65,000 patients, representing a small yet notably sick and complex patient group, primarily composed of individuals from middle- and high-income groups. In contrast, most patients in Karachi depend on lower-cost government or neighborhood private health facilities for emergency care. The numbers seen in the ED decreased significantly during the COVID-19 pandemic. The care in the ED is provided by emergency medicine trainees/residents under the supervision of attendings/consultants 24/7. On arrival at the ED, triage nurses prioritize and document vital signs on all patients using the Emergency Severity Index (ESI) [24]. Those who are too unstable to wait at triage are brought directly to the resuscitation room as per the institutional protocol. Triage information, including vital signs, laboratory results, radiology reports, and pharmacy orders, are electronically recorded, while physician and nursing assessments are documented manually in paper files.

### Participants

This study included all patients aged 18 years or older with triage systolic blood pressure readings of ≥180 mm Hg or diastolic blood pressure readings of ≥120 mm Hg. Only single blood pressure reading was taken at triage as obtaining multiple blood pressure readings for each patient during their initial assessment might introduce delays in patient care and require significantly increased resources. We excluded patients <18 years of age, pregnant women, and those who did not consent to participate or were triaged but admitted to the hospital without treatment in the ED. Pregnant patients were excluded as they followed a different care pathway in the ED. Written consent was obtained from all eligible patients or their next of kin. The data presented herein constitutes a segment of an intervention study designed to assess the impact of implementing an ED checklist on the practices and outcomes of patients presenting with acute severe hypertension. The primary findings from the intervention study will be detailed in a separate publication.

### Data collection

Trained Research Assistants (RAs) conducted the 24/7 prospective enrollment of patients, identifying eligible individuals through electronically available triage vital signs. Data were not collected for 28 days due to public/religious holidays and during the initial 4 months and 12 days of the COVID-19 pandemic when all research was temporarily stopped to respond to the pandemic. Upon identifying a patient through triage records, the RAs introduced themselves to the patients or their families, explained the study’s purpose, answered questions, obtained written consent, and confirmed follow-up contact information. During the peak of the COVID-19 pandemic, verbal telephone-based consent was obtained in adherence to institutional guidelines and Institutional Review Board (IRB) approval.

RAs systematically collected demographic and clinical data during the ED visit, utilizing ED medical records for participants who consented to participate in the study. The clinical course was extracted from medical charts using study-specific data collection forms for patients admitted to the hospital. Patient outcomes at 1, 3, and 6 months were gathered prospectively via a structured questionnaire during telephonic interviews. All data was entered into the Research Electronic Data Capture (RED-Cap) [25].

To ensure data accuracy and consistency, all RAs underwent comprehensive training before data collection, covering study implementation processes and data collection procedures and using RED-Cap’s data entry functions. RAs were not blinded to the outcomes. A refresher training session addressed any issues encountered during data collection or entry. A project coordinator’s daily audit of all data forms served as a quality control measure.

After discharge from the ED or inpatient units, RAs called each patient at 1, 3, and 6 months post-discharge using the provided telephone numbers. To minimize loss to follow-up during telephonic follow-ups, RAs called patients three times on day 1, twice on days 2 and 3, and once each from days 4 to 14. Patients were deemed lost to follow-up if contact was not established within 14 days.

To mitigate the risk of over-diagnosing HE, each patient’s medical record underwent a thorough review by one of the two Emergency Medicine faculty members who were residency-trained and worked as supervising clinicians in the ED. The goal was to identify individuals with preexisting chronic kidney disease or a history of prior stroke to avoid misclassifying them as having a new end-organ damage as thus over-estimating the rates of HE. This was done through a joint review of medical records through in-person meetings by RAs and faculty members. Both Emergency Medicine faculty members were integral members of the study team and were familiar with the case definition as well as the inclusion and exclusion criteria. It is important to note that they were not blinded to the study objectives, and we did not formally assess the interrater reliability or agreement.

### Study measures

HE was defined as triage SBP ≥180 mmHg or DBP ≥120 mm Hg associated with evidence of any of the following: hypertensive heart failure, cardiac ischemia evident by complaint of typical chest pain or angina equivalent symptoms associated with new electrocardiogram changes or elevated troponins, transient ischemic attack, hypertensive encephalopathy, subarachnoid hemorrhage, ischemic or hemorrhagic stroke, acute kidney injury as per the Kidney Disease Improving Global Outcome-KDIGO criteria, grade 3 to 4 hypertensive retinopathy and aortic dissection. Specifically, the KDIGO criteria (increase in serum creatinine of ≥ 0.3mg/dl within 48hrs; or increase in serum creatinine to ≥ 1.5 times of baseline which is known or presumed to have occurred within the prior 7 days; or urine volume <0.5 ml/kg/h for 6 hours) was used to define AKI [26]. Patients with SBP ≥180 mmHg or DBP ≥120 mm Hg at triage and no evidence of end-organ damage were labeled as HU. During the ED visit, we obtained data on age, gender, triage category, presenting complaint, comorbidities, use of antihypertensive medications and BP at triage, first BP when the patient was brought in the treatment area and a BP at the time of discharge or admission to the hospital. During telephone follow-ups, we collected data on outcomes such as death, readmissions to hospitals, frequency of monitoring of blood pressure (on a seven-point scale from daily BP checks to rarely), adherence to antihypertensive medications (yes vs. no), and follow-up with the primary physicians (three possible responses; visited once after discharge, visited twice after discharge, visited more than twice after discharge) (S1 File).

### Statistical analysis

All data were collected on (REDCap) and analyzed using Stata-14 for statistical analysis [27]. After checking normality assumptions, descriptive statistics (mean, standard deviation, minimum, maximum, numbers, and percentage) were reported for continuous variables. We calculated frequencies for categorical variables and applied Kaplan-Meier plots to visualize the survival patterns. After selecting confounding indicators for the study, we did the univariate analysis, taking mortality as a dependent variable and selecting significant variables in the univariate model. Independent variables with a p-value of <0.2 were used in a multiple survival Cox regression model. We built multivariate models using the stepwise backward selection. The first set of models included all risk factors up to discharge from the hospital (e.g., male gender, age > 65, acute myocardial infarction, etc.)The second set of models included all risk factors till the follow-up (e.g., lack of home blood pressure monitoring, non-adherence to antihypertensive medications, lack of regular follow-up with primary care physician). Only variables with a p-value of ≤ 0.05 were kept in the model. The results were reported as relative risk (RR) with a 95% confidence interval. We performed sensitivity analysis where applicable, e.g., for HE and HU patients separately.

Ethics approval for the study was obtained by IRBs at the Aga Khan University and Johns Hopkins University.

## Results

**Fig 1:**
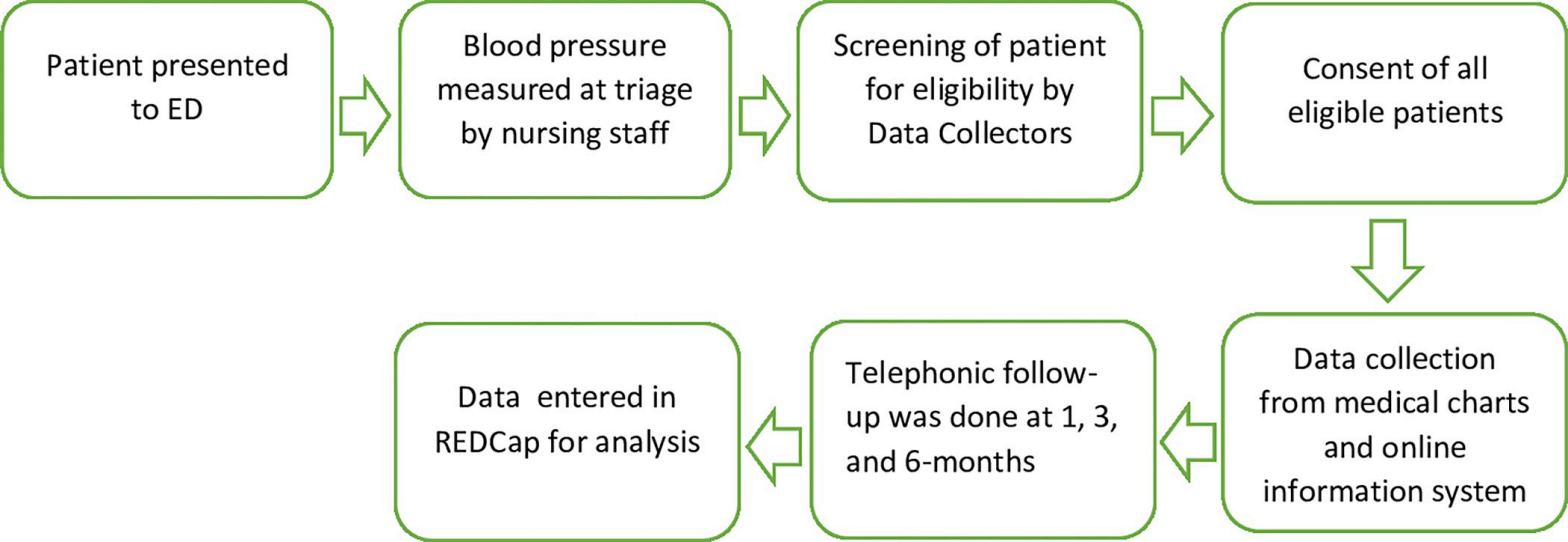
Patient enrollment and data collection information.

During the study period, 49,431 adult patients visited the ED, with triage vitals available for 40,873. Among them, 1,525 patients (3.7%) met the inclusion criteria. Due to the limited availability of RAs during holidays and some weekends, we approached 1270 patients, of which 1,164 patients (91.6%) provided consent for participation. Of these, two left ED after triage, and one was admitted directly to the hospital without any further ED management. The final cohort included 356 patients (30.6%) diagnosed with HE and 805 (69.2%) with HU. Six-month follow-up data was available for 1,065 patients (91.7%), with 326 (91.6%) in the HE and 739 (91.8%) in the HU group (Fig 1).

The mean age of the patients was 60.4 years (±14.33). Over half of the patients were females (n = 680, 59%), and 1007 (86.7%) were triaged as priority 1 and 2 on the ESI triage scale. The most common presenting complaint was nausea and vomiting (n = 177, 15.2%), followed by abdominal pain (n = 174, 15%) and chest pain (n = 143, 12.3%). Around 108 patients (9.1%) were asymptomatic at presentation. Most patients had a past medical history of hypertension (n = 972, 83.7%), and half had a history of diabetes (n = 569, 49%). Only 438 patients (37.7%) were taking antihypertensive medications. The most common end-organ damage was kidney (n = 153, 13.1%) followed by brain (n = 115, 9.9%) and heart (n = 109, 9.3%) (Table 1).

**Table 1:** Demographic and baseline health characteristics, and new-onset end-organ damage of the sample population (n = 1161).

Characteristics	Results
Age in years *(n*, *%)*	
18-34	52 (4.5)
35-64	615 (53)
≥65	494 (42.5)
Gender *(n*, *%)*	
Male	481 (41.4)
Female	680 (58.6)
Triage Category *(n*, *%)*	
-ESI 1	377 (32.5)
ESI 2	630 (54.3)
ESI 3	154 (13.3)
Presenting complaints *(n*, *%)* *	
Nausea and vomiting	177 (15.2)
Abdominal pain	174 (15)
Chest pain	143 (12.3)
Shortness of breath	142 (12.2)
Anxiety or vague discomfort (*Ghabrahat* in the Urdu language)	120 (10.3)
High blood pressure	108 (9.3)
Unilateral limb weakness	92 (7.9)
Headache	87 (7.5)
Injury/Accident/Fall	86 (7.4)
Altered mental state	80 (6.9)
Vertigo	68 (5.9)
Fever	63 (5.4)
Pain	61 (5.2)
Generalized weakness	46 (3.9)
Dysuria/Burning micturition	43 (3.7)
Constipation/Diarrhea	39 (3.3)
Others^†^	109 (9.3)
Comorbidities *(n*, *%)*	
Hypertension	972 (83.7)
Diabetes	569 (49.0)
Ischemic heart disease	213 (18.3)
Chronic kidney disease	121 (10.4)
Cerebrovascular accident	91 (7.8)
History of surgery	53 (12)
Cancer	52 (4.47)
Others ^‡^	210 (18.0)
Patients taking antihypertensive medications	438 (37.7)
End organ damage (EOD) *(n*, *%)*	
Kidney	153 (13.1)
Brain	115 (9.9)
Heart	109 (9.3)

* Patient had the option of providing multiple responses.

† Slurring of speech, facial deviation/ swelling, GI/GU bleeding, epistaxis, decreased urine output, blurred vision, bilateral leg swelling, each under 2% of the total presenting chief complaints.

‡ Musculoskeletal disorders, asthma, thyroid disorders, chronic liver disease, dyslipidemia, smoking, psychiatric disorders, neurological disorders, hepatitis, chronic pulmonary obstructive pulmonary disease, overweight/ obesity, blood disorders, eye, interstitial lung disease, congenital disease each under 2% of all self-reported comorbidities

ESI: Emergency Severity Index

Regarding the outcomes, 52 patients (14.6%) with HE had passed away within the six months. Among these, two patients died in the ED, and eight during hospital admission. A significant portion of deaths occurred within the initial month (38.4%), followed by 21.1% in the second and third months, and another 21.1% in months 4-6 post-index visits.

**Fig 2:**
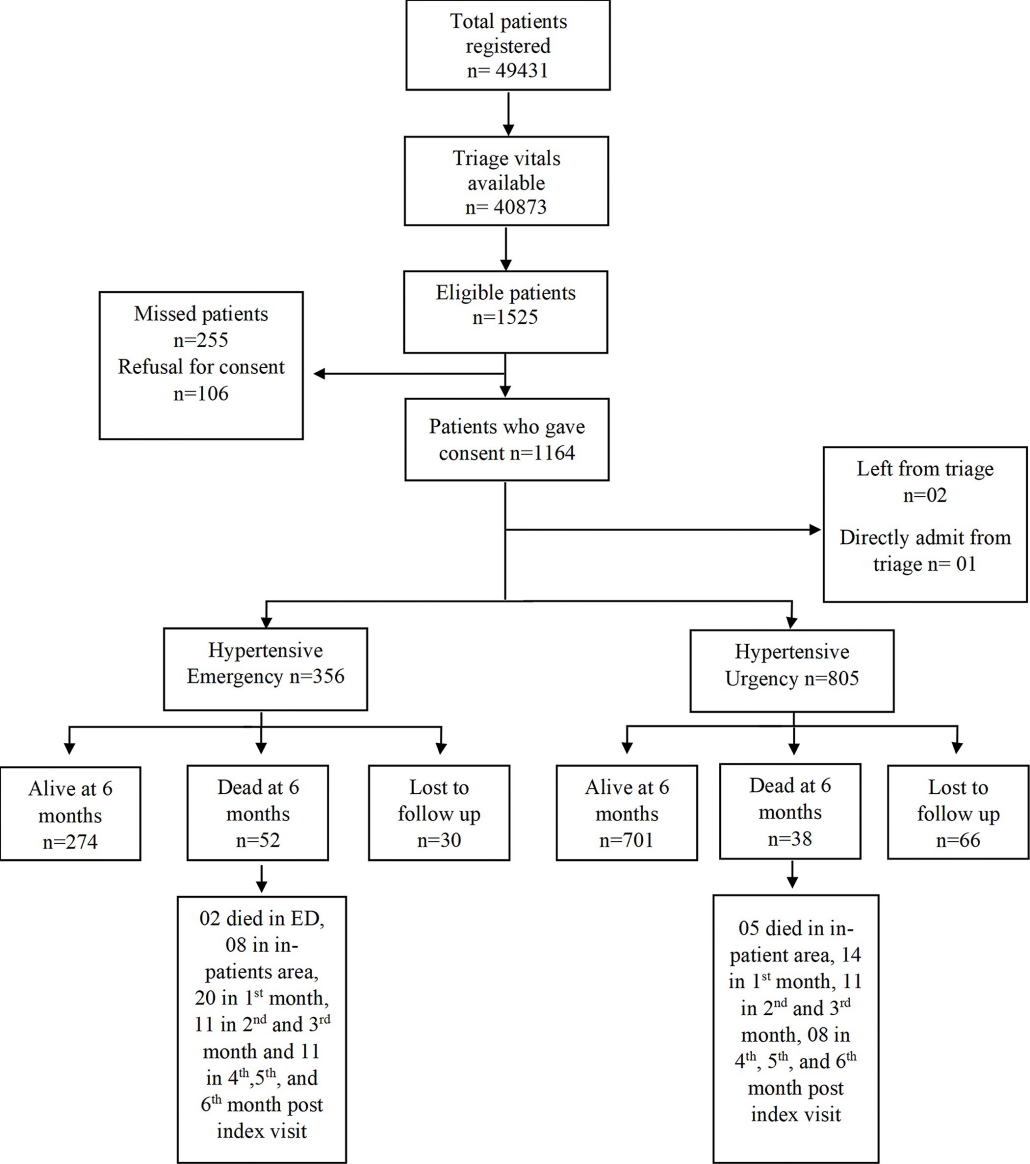
Patient distribution and outcomes.

In contrast, among patients with HU, 701 (87.0%) remained alive, while 38 (4.7%) had died and 66 (8.3%) were lost to follow-up. The majority of HU-related deaths occurred in the first-month post-discharge (36.8%), with additional occurrences between 1-3 months (28.9%) and 4-6 months (21.0%) post-index visits (Fig 2).

Patients with HE had a three times higher risk of death than those with HU. Additionally, HE patients had a higher readmission rate (17.0%) compared to HU patients (11.6%). The primary causes of readmission included raised blood pressure (20.8%), acute myocardial infarction (7.1%), and kidney failure (9.4%).

More than 85% (n = 985) of all follow-up patients self-reported regularly monitored their blood pressure, and 84.1% (n = 930) of the patients also reported to be adherent with their antihypertensive medications prescribed at the time of discharge. The average medication adherence rate increased from 1^st^ month to 3^rd^ month for both HE and HU groups (83.7% to 93% and 87.4% to 93.9%). However, only 19.9% (n = 220) of the patients followed up with their primary physician during the six-month follow-up period (Table 2).

**Table 2:** Cumulative outcomes at six months follow-up.

Outcomes	1-month follow-up *		3 months follow-up ^†^		6 months follow-up ^‡^	
	N = 1051		N = 1024		N = 994	
	HE	HU	HE	HU	HE	HU
	310 (30.0%)	741 (70.0%)	301 (29.3%)	723 (70.7%)	285 (28.6%)	709 (71.4%)
Checking BP regularly (n, %)	277 (83.7)	674 (87.4)	280 (93.0)	679 (93.9)	266 (93.3)	661 (93.2)
Taking antihypertensive medications regularly (n, %)	270 (87.1)	602 (81.2)	268 (89.0)	587 (81.2)	255 (89.5)	563 (79.4)
Followed-up with a primary physician (n, %)	88 (28.4)	177 (23.9)	83 (27.6)	180 (24.9)	76 (26.7)	150 (21.2)
Required admission to hospital (n, %)	29 (9.4)	31 (4.2)	29 (9.6)	44 (6.1)	27 (9.5)	44 (6.2)
Death (n, %)	20 (6.5)	14 (1.9)	11 (3.7)	11 (1.5)	11 (3.9)	8 (1.1)

* 8.3% (n = 95) lost to follow-up at 1 month.

† 10.9% (n = 122) lost to follow-up at 3 months.

^‡^ 13.9% (n = 152) lost to follow-up at 6 months.

Univariate Cox regression analyses revealed a higher mortality risk for those aged 65 or older, with acute myocardial infarction, acute kidney injury, stroke, ED length of stay >6 hours, and a hospital stay >48 hours. Regular blood pressure monitoring, following up with a primary care physician, and adhering to antihypertensive medications were associated with reduced mortality risk (Table 3 and S1 Fig). Multivariable Cox regression identified age >65, stroke, acute kidney injury, non-monitored bed admission, and hospital stay >48 hours as predictors of higher mortality. Regular blood pressure monitoring and adherence to antihypertensive medications were associated with significantly lower mortality risk. Sensitivity analyses showed similar trends in HE and HU patients (Table 3).

**Table 3:** Univariate and multivariate Cox regression analysis for mortality.

Factors before discharge	Mortality at six months		Univariate	Multivariate*	Multivariate†
	n	%	RR [95% CI]	RR [95% CI]	RR [95% CI]
Male gender	35	8	1.23 [0.78 -1.94]	-	-
Age ≥ 65 years	43	10	2.22 [1.41 -3.50]	1.90 [1.20 to 3.02]	-
Acute myocardial infarction	13	12	2.84 [1.63 -4.81]	-	-
Acute Kidney Injury	22	15	2.67 [1.62 -4.40]	1.82 [1.09 to 3.04]	-
Stroke	13	18	2.84 [1.67 -4.83]	2.09 [1.21 to 3.61]	-
Admit to intensive care / special care	18	16	1.11 [0.66 -1.89]	-	-
Admission to ward	45	15	4.27 [2.69 -6.78]	2.50 [1.48 to 4.24]	-
Length of hospital stay ≥ 48 hours	51	13	4.11 [2.53 -6.69]	1.95 [1.12 to 3.42]	-
Length of ER Stay ≥ 6 hours	51	9	2.05 [1.26 -3.34]	-	-
**Factors after discharge**					
Readmission	9	6	0.84 [0.42-1.69]	-	-
Regular monitoring of blood pressure	16	2	0.03 [0.01-0.04]	-	0.08 [0.03-0.19]
Followed up with primary care physician	2	1	0.11 [0.03-0.44]	-	
Taking antihypertensive medications regularly	15	2	0.04 [0.02-0.07]	-	0.23 [0.09-0.56]

* Risk factors prior to discharge

† Risk factors after discharge

The risks for mortality were much lower for those who regularly monitored their blood pressure (aRR = 0.08, 95%CI = 0.03-0.19) or reported adherence to antihypertensive medications (aRR = 0.23, 95%CI = 0.09-0.56) (Table 3). Sensitivity analyses showed similar trends in HE and HU patients (not shown as associations were not significant because of underpowered sample sizes).

## Discussion

Our study reveals a remarkably high prevalence of Acute Severe Hypertension (ASH) in patients presenting to the study ED in Karachi, Pakistan. Of concern were the elevated six-month admission and mortality rates, observed not only among individuals with HE but also among patients with HU. Notably, one in six patients with HE and one in twenty with HU had succumbed during the six-month follow-up period. These findings underscore the pronounced short-term morbidity and mortality risks associated with hypertensive emergencies and urgencies, highlighting the critical importance of regular blood pressure monitoring and strict adherence to antihypertensive medications. This bears particular significance in the context of Pakistan, wheremore than 70% of the hypertensive population receiving treatment exhibits poorly controlled blood pressure [28].

The prevalence of Acute Severe Hypertension (ASH) among ED patients exhibits substantial global variation. In our study, which utilized triage blood pressure readings for patient inclusion, we identified a notable ASH prevalence of 37.3 per 1000 cases. This contrasts with reported rates ranging from 58 per 1000 ED visits in South Korea [17] to 0.64 per 1000 in Thailand [22]. The staggering 100-fold difference is unlikely to reflect a genuine discrepancy in prevalence across the general population in these regions but rather suggests methodological variations in the studies. These differences encompass factors such as the timing of blood pressure measurements during ED care, type of hospital, inclusion criteria, and patient demographics, particularly the average age of the ED population. Importantly, our study was conducted in a major referral center known for managing patients referred from other regional hospitals due to the complexity and acuity of illness, which may have potentially amplified the observed prevalence. To some extent, these differences also highlight a significant gap in hypertension management in primary care in different settings [29]. Addressing these discrepancies is crucial for a comprehensive understanding of ASH prevalence and ensuring effective hypertension management strategies across diverse healthcare settings.

We found a concerning six-month mortality rate amongst patients with ASH, including those with HU. In fact, in our population, mortality associated with HU was about 12 times the expected mortality in the general population [30]. Like prevalence, mortality rates across diverse settings exhibit significant variation due to differences in case definitions, follow-up duration, average age, preexisting morbidity, and the quality and availability of post-acute care in both inpatient and outpatient settings [15, 16, 18–20, 23]. Our inpatient mortality from HE aligns with high-resource settings [7, 19] and notably falls below reported rates in low-resource settings [13, 29]. Importantly, increased mortality in our population occurs during the post-discharge phase, emphasizing the potential impact of frequency of close post-discharge follow-up and the quality of care during the follow-up including inducement and encouragement regarding lifestyle changes and management of other cardiovascular risk factors using well-known evidence-based interventions [31]. We observed a higher mortality rate among patients admitted to the non-monitored bed. This was likely influenced by families choosing to restrict resuscitation measures for patients with low prospects of meaningful survival. Furthermore, AKUH is a private setup and individuals are required to pay directly for medical services. The increased financial burden associated with ICU care may influence families’ decisions regarding the level of care provided to their loved ones, particularly if they perceive limited prospects for meaningful recovery.

Our study carries significant implications for emergency care delivery, system design, and future research. Firstly, aligning with a growing body of literature, we assert that ED visits for patients with hypertensive urgencies represent a pivotal opportunity to identify high-risk individuals and intervene to prevent catastrophic outcomes [10, 21, 32, 33]. Achieving this necessitates the improved integration of EDs and primary care services through the adaptation of proven active and passive mechanisms. These include establishing ED-based follow-up clinics, implementing rapid scheduling of appointments for discharged patients [34], deploying care navigators to facilitate identification and referral to appropriate primary care settings [35], and using community resources such as community health workers [36]. Once engaged with primary care providers, there is a need to advocate for practical and feasible lifestyle modifications, particularly for individuals residing in high-density urban environments, while also recognizing and aggressively addressing cardiovascular complications [37].

For the research community, achieving harmonization in ED-based diagnoses and case definitions is essential for advancing the field. While discarding the term "Hypertensive Urgency" is appropriate to prevent aggressive blood pressure lowering in the acute care setting, it remains crucial to emphasize that the condition is urgent, requiring close outpatient follow-up. Further exploration is warranted to understand barriers hindering the successful transition of care, encompassing factors such as cost, transportation, and patient awareness. Additionally, elucidating the role of newer options, particularly telemedicine, holds significance, especially for individuals with hypertensive urgencies, and combining them with the traditional role of community health workers who could assist with ensuring follow-up with primary care and sustainable lifestyle changes.

Lastly, given the constraints of critical care resources, there is merit in sub-categorizing patients with hypertensive emergencies who can be managed in alternative settings outside of an intensive care unit without worsening outcomes. This categorization could serve as a valuable guide for resource allocation, optimizing patient care in situations where critical care resources are limited.

Our study is subject to several limitations. Firstly, being a single-center study based at a fee-for-service, referral center, it likely represents a sicker and relatively higher-income urban population. This may not fully capture the socio-economic diversity across urban and rural areas of Pakistan. Nevertheless, given the relatively privileged socio-economic group seen in this ED, we suspect that rates of undiagnosed and untreated hypertension and challenges in ensuring follow-up are likely higher in less privileged populations.

Secondly, our follow-up data on compliance with BP measurement and medication adherence were self-reportedand appear to be higher than reported for the general population [38]. However, only 21-28% of the patients followed up with their primary physician. In Pakistan, medications are readily available without prescription at pharmacies which could explain the disparity between follow-up with doctors and medication usage. Moreover, these patients had a perceived life-threatening event requiring an ED visit which could have played a role in higher compliance. Additionally, we observed an increase in self-reported compliance with BP home monitoring and medication adherence during subsequent calls, possibly influenced by the Hawthorne effect or patients’ over-reporting for fear of judgment by the interviewers or social desirability bias [39–41]. Also, we recognizethat the interpretation of “taking medications regularly” and "regularly monitored blood pressure" may vary among patients. The term "regularly" may have been subject to individual interpretation, leading to potential misclassification bias in self-reported responses.

Finally, due to the limitation of telephone conversations, we opted to limit the call duration and only collect data on rates of death, and self-reported causes of death without getting details surrounding the circumstances of death. This limitation hinders the direct attribution of death to cardiovascular complications and, consequently, the assessment of the preventability of these deaths. The study identifies potential health system weaknesses, but further specific information elucidating the breaks in the care pathway and understanding the circumstances surrounding readmission and death need to be investigated. Additionally, we acknowledged the occurrence of COVID-19 during the study, comparing mortality before and during the pandemic, revealing no significant differences between the two populations. Nevertheless, the elevated all-cause mortality and readmission rates underscore the necessity for a more profound understanding of the circumstances surrounding readmission and death.

## Conclusion

In summary, our investigation revealed a high prevalence of acute severe hypertensionin our patient cohort-, concomitant with increased six-month mortality and hospitalization rates. This emphasizes the ED visit for Acute Severe Hypertension (ASH) as a potential critical opportunity for averting both short-term and longer-term health complications. The results underscore the significance of a more in-depth exploration of the determinants leading to poor outcomes and advocate for the implementation of established interventions for outpatient management of hypertension among patients discharged from ED.

## Supporting information

S1 Fig Survival outcomes among different subgroups. (TIFF)

S1 File (PDF)
