# Supplementary figures and images for: Assessing the impact of acute severe hypertension in the emergency department: A prospective cohort study in Karachi, Pakistan
Source: PLOS Glob Public Health. 2024 Dec 4;4(12):e0003948. doi: 10.1371/journal.pgph.0003948 (PMC11616827; doi:10.1371/journal.pgph.0003948)

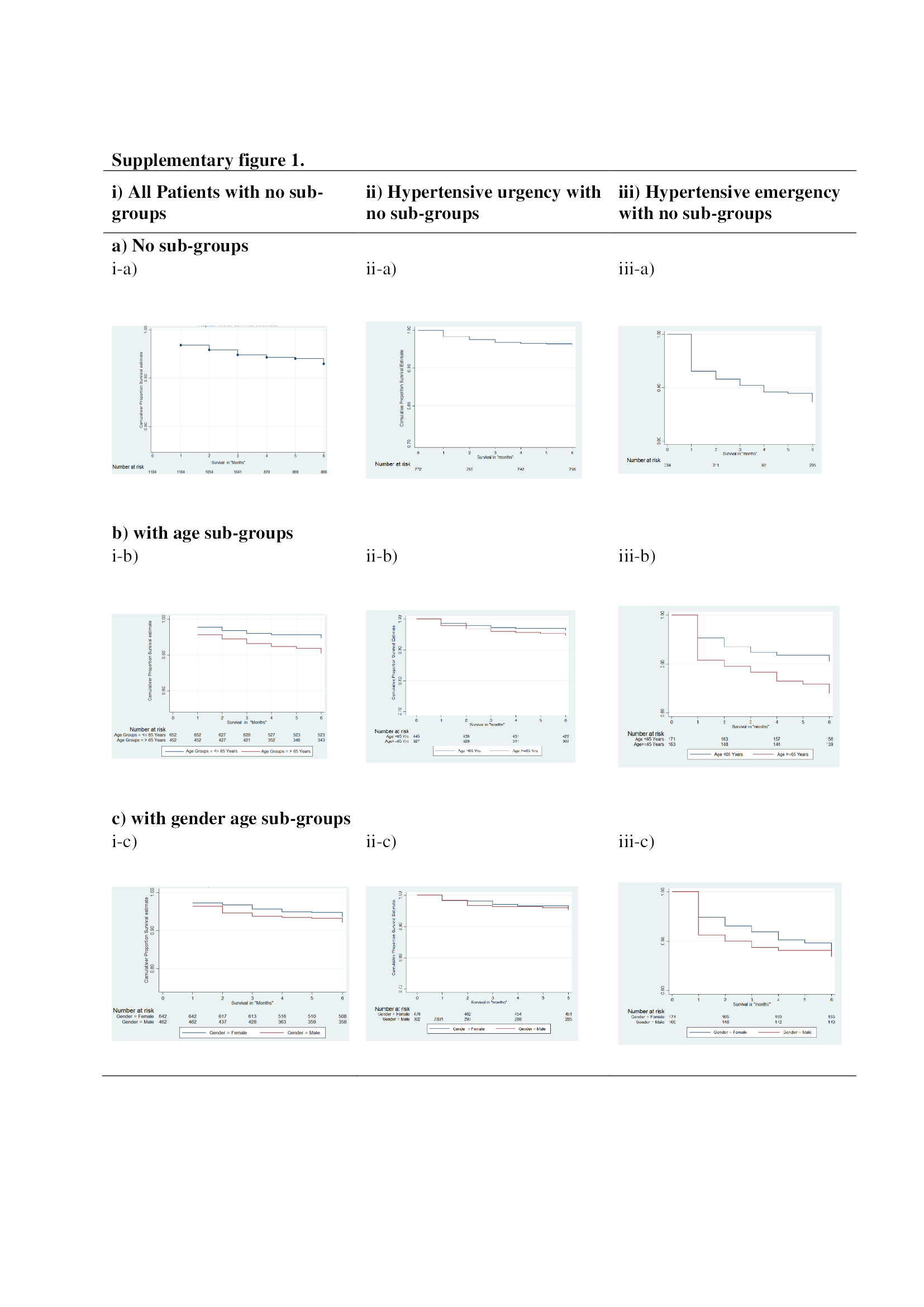

Supplement: S1 Fig — (TIFF) [file pgph.0003948.s001.tiff]
