# Supplementary material for: Assessing the impact of acute severe hypertension in the emergency department: A prospective cohort study in Karachi, Pakistan
Source: PLOS Glob Public Health. 2024 Dec 4;4(12):e0003948. doi: 10.1371/journal.pgph.0003948 (PMC11616827; doi:10.1371/journal.pgph.0003948)
Supplement: S1 File — (PDF) [file pgph.0003948.s002.pdf]

# Triage history

| | |
|---|---|
| Patient ID | ______________________ |
| Date of triage. | ______________________ |
| Time of triage | ______________________ |
| Triage Category | ○ P1<br>○ P2<br>○ P3 |
| Sex of the patient | ○ Male<br>○ Female |
| Systolic Blood pressure | ______________________<br>((in mm of Hg)) |
| Diastolic Blood Pressure | ______________________<br>((in mm of Hg)) |
| Pulse | ______________________<br>((in beats/min)) |
| Temperature<br>(if patient is afebrile, please input 37.8 degrees Celsius) | ______________________<br>((in degree celsius) ) |
| Respiratory rate | ______________________<br>((in breaths/min)) |
| Oxygen saturation | ______________________<br>((%)) |
| Date of birth of the patient? | ______________________ |
| Age of the patient | ______________________ |

Chief complaint

- ☐ Chest pain, chest pressure, chest tightness
- ☐ Shortness of breath
- ☐ Headache
- ☐ Vertigo
- ☐ Nausea or vomiting
- ☐ Abdominal pain
- ☐ Discomfort (ghabrahat)
- ☐ Blurred vision
- ☐ Bilateral leg swelling
- ☐ Slurring of speech
- ☐ Facial deviation
- ☐ Altered mental state
- ☐ Decreased urine output
- ☐ Any unilateral / one sided limb weakness
- ☐ No symptoms (with high BP)
- ☐ Others

Chief complaint, others (Specify)

__________________________________

If pain was specified in any region, was the pain severity score taken?

- ◯ Yes
- ◯ Not documented

What is the severity of the pain?

__________________________________
((please enter a number between 0 to 10))

Did the patient receive any analgesia for the pain?

◯ Yes ◯ No ◯ Not documented

## What analgesics did the patient receive?

| | Yes, Oral | Yes, IV | No |
|---|---|---|---|
| Opioids (Tramadol, Nalbuphine/Kinz) | ☐ | ☐ | ☐ |
| Paracetamol | ☐ | ☐ | ☐ |
| NSAIDS (Ketorolac, Naproxen, Diclofenac, Brufen) | ☐ | ☐ | ☐ |
| Others | ☐ | ☐ | ☐ |

Others (Specify)

__________________________________

# Assessments and examinations

What is the Systolic blood pressure at assigned bed?

______________________
((in mm/Hg))

What is the diastolic blood pressure at assigned bed?

______________________
((in mm of Hg))

What was the pulse at assigned bed?

______________________
((in beats/min))

What was the temperature (in degree Celsius) at assigned bed?

______________________
((in degree celsius) )

What was the respiratory rate at assigned bed?

______________________
((in beats/min))

What was the oxygen saturation at assigned bed?

______________________
((%))

On CNS exam, were any new focal deficits found?
- ◯ Yes
- ◯ No
- ◯ Not documented

Was the GSC taken?
- ◯ Yes
- ◯ Not documented

What was the GCS?

______________________

On Chest exam, were any bilateral crackles heard?
- ◯ Yes
- ◯ No
- ◯ Not documented

On CVS exam. was the jugular venous pressure raised?
- ◯ Yes
- ◯ No
- ◯ Not documented

On CVS exam, were additional heart sounds heard or an S3 gallop heard?
- ◯ Yes
- ◯ No
- ◯ Not documented

On a CVS exam, did the patient have pedal edema?
- ◯ Yes
- ◯ No
- ◯ Not documented

Was a fundoscopy exam performed?
- ◯ Yes
- ◯ Not documented

Was papilledema noticed on the fundoscopy?

◯ Yes
◯ No
◯ Not documented

# Past Medical History

Does the patient have any co-morbidities?
○ Yes
○ No
○ Not documented

## Please select the co-morbid conditions that the patient has.

| | Yes | No | 99 Not documented |
|---|---|---|---|
| Diabetes Mellitus | ○ | ○ | ○ |
| Hypertension | ○ | ○ | ○ |
| Cerebrovascular accident/ stroke | ○ | ○ | ○ |
| Ischemic Heart disease | ○ | ○ | ○ |
| Asthma | ○ | ○ | ○ |
| Chronic obstructive pulmonary disease | ○ | ○ | ○ |
| Interstitial lung disease | ○ | ○ | ○ |
| Chronic liver disease | ○ | ○ | ○ |
| Dyslipidemia | ○ | ○ | ○ |
| Chronic kidney disease | ○ | ○ | ○ |
| Overweight/ obesity | ○ | ○ | ○ |
| Smoking/exposed to second hand smoking | ○ | ○ | ○ |
| Thyroid disorders (Hyper and hypothyroidism) | ○ | ○ | ○ |
| Hepatitis B | ○ | ○ | ○ |
| Hepatitis C | ○ | ○ | ○ |
| Others | ○ | ○ | ○ |

"Others"
(Please specify) ______________________________

Is the patient taking any anti-hypertensive medications or being prescribed any anti-hypertensive medications?
○ Yes
○ No
○ Not documented

## What anti-hypertensive medications is the patient taking or being prescribed?

| | Yes | No | Not documented |
|---|---|---|---|
| Angiotensin converting enzyme (ACE) inhibitors (captopril, enalapril, lisinopril) | ○ | ○ | ○ |
| Angiotensin II Receptor Blockers (ARB) (candesartan, valsartan, losartan, erbesartan) | ○ | ○ | ○ |

| | | | |
|---|---|---|---|
| Calcium Channel Blockers (verapamil, amlodipine, nefedipine, diltiazem,) | ◯ | ◯ | ◯ |
| Beta Blockers (atenolol, propranolol, metoprolol, carvedilol, labetolol) | ◯ | ◯ | ◯ |
| Hydralazine | ◯ | ◯ | ◯ |
| Diuretics (furosemide - Lasix, spironolactone, hydrochlorothiazide) | ◯ | ◯ | ◯ |

---

Is the patient compliant to anti-hypertensive medications?
(taken within 24 hours)

◯ Yes ◯ No ◯ Not documented

# Diagnostic tests

| | |
|---|---|
| Creatinine? | ◯ Yes<br>◯ No |
| Creatinine value | __________________________ |
| CBC? | ◯ Yes<br>◯ No |
| Hemoglobin value | __________________________ |
| Hematocrit value | __________________________ |
| Platelet count value | __________________________ |
| BUN? | ◯ Yes<br>◯ No |
| BUN value | __________________________ |
| Electrolytes - Sodium (Na)? | ◯ Yes<br>◯ No |
| Sodium value | __________________________ |
| Electrolyte - Potassium (K)? | ◯ Yes<br>◯ No |
| Potassium value | __________________________ |
| Electrolyte - Chloride? | ◯ Yes<br>◯ No |
| Chloride value | __________________________ |
| Electrolyte - bicarbonate (HCO3)? | ◯ Yes<br>◯ No |
| Bicarbonate value | __________________________ |
| Coagulation profile - PT/ INR? | ◯ Yes<br>◯ No |

| | |
|---|---|
| Prothrombin time | __________________________________ |
| INR | __________________________________ |
| Coagulation profile - APTT? | ◯ Yes<br>◯ No |
| Activated partial thromboplastin time | __________________________________ |
| 1st troponin test? | ◯ Yes<br>◯ No |
| 1st troponin value? | __________________________________ |
| 2nd troponin test | ◯ Yes<br>◯ No |
| 2nd troponin value? | __________________________________ |
| ECG performed? | ◯ Yes<br>◯ Not found |

| | Yes | No |
|---|---|---|
| ST-T changes | ◯ | ◯ |
| LVH | ◯ | ◯ |

| | |
|---|---|
| Urine detail report performed? | ◯ Yes<br>◯ No |
| Did the urine analysis show proteinuria? | ◯ Yes<br>◯ No/ Negative |
| Urine Protein? | __________________________________ |
| Did urine analysis show hematuria? | ◯ Yes<br>◯ No/Nil |
| Hemoglobin in urine? | __________________________________ |
| RBCs in urine? | __________________________________ |
| Was chest xray ordered? | ◯ Yes<br>◯ No |

| | Yes | No |
|---|---|---|
| Normal/unremarkable | ◯ | ◯ |
| Signs of fluid overload (Hilar congestion, pulmonary edema, upper lobe diversion, pulmonary vascular congestion) | ◯ | ◯ |
| Cardiomegaly | ◯ | ◯ |

Was a CT scan of the head performed?
◯ Yes
◯ No

| | Yes | No |
|---|---|---|
| Normal | ◯ | ◯ |
| Infarction | ◯ | ◯ |
| Bleeding | ◯ | ◯ |

Was an MRI of the brain/head performed?
◯ Yes
◯ No

| | Yes | No |
|---|---|---|
| Normal | ◯ | ◯ |
| Infarction | ◯ | ◯ |
| Bleeding | ◯ | ◯ |

# End organ damage

Was a diagnosis of end-organ damage made/ found?

◯ Yes
◯ No
◯ Not documented

## End organ damage details

| | Yes | No | Not documented |
|---|---|---|---|
| Heart (trop positive OR ST-T changes in ECG) | ◯ | ◯ | ◯ |
| Kidney (Proteinuria, raised creatinine) | ◯ | ◯ | ◯ |
| Eye (Papilledema) | ◯ | ◯ | ◯ |
| Brain (Stroke, sub-arachnoid hemorrhage, Hypertensive encephalopathy) | ◯ | ◯ | ◯ |

# Medication/ treatment/ interventions provided during stay

| | |
|---|---|
| Were Per oral (PO) medications administered? | ◯ Yes<br>◯ No<br>◯ Not documented |
| Angiotensin converting enzyme (ACE) inhibitors (captopril, enalapril, lisinopril) | ◯ Yes<br>◯ No<br>◯ Not documented |
| Angiotensin converting enzyme dose? | __________________________ |
| Angiotensin II Receptor blockers (ARB) (candesartan, valsartan, losartan, erbesartan) | ◯ Yes<br>◯ No<br>◯ Not documented |
| Angiotensin II receptor blockers dose? | __________________________ |
| Calcium channel blockers (Verapamil, amlodipine, nifedipine, diltiazem) | ◯ Yes<br>◯ No<br>◯ Not documented |
| Calcium channel blockers dose? | __________________________ |
| Beta Blockers (atenolol, propranolol, metoprolol, carvedilol, labetalol) | ◯ Yes<br>◯ No<br>◯ Not documented |
| Beta blockers dose? | __________________________ |
| Hydralazine | ◯ Yes<br>◯ No<br>◯ Not documented |
| Hydralazine dose? | __________________________ |
| Diuretics (furosemide - lasix, spironolactone, hydrochlorothiazide) | ◯ Yes<br>◯ No<br>◯ Not documented |
| Diuretics dose? | __________________________ |
| Were Intravenous (IV) medications administered? | ◯ Yes<br>◯ No<br>◯ Not documented |

| | |
|---|---|
| Beta blockers (metoprolol, labetalol) | ◯ Yes<br>◯ No<br>◯ Not documented |
| Beta blockers dose? | __________________________________ |
| Calcium channel blockers (Verapamil, Diltiazem) | ◯ Yes<br>◯ No<br>◯ Not documented |
| Calcium channel blockers dose? | __________________________________ |
| Hydralazine | ◯ Yes<br>◯ No<br>◯ Not documented |
| Hydralazine dose? | __________________________________ |
| Diuretics (furosemide - Lasix, spironolactone, hydrochlorothiazide) | ◯ Yes<br>◯ No<br>◯ Not documented |
| Diuretics dose | __________________________________ |
| Infusion started | ◯ Yes<br>◯ No<br>◯ Not documented |
| Nitrates (nitroprusside and glyceryl trinitrate (GTN)) | ◯ Yes<br>◯ No<br>◯ Not documented |
| Nitrate infusion dose? | __________________________________ |
| Labetalol infusion | ◯ Yes<br>◯ No<br>◯ Not documented |
| Labetalol infusion dose? | __________________________________ |

# ER discharge/ disposition information

| | |
|---|---|
| ER diagnosis? | __________________________ |
| ER disposition? | ◯ Admitted<br>◯ Discharged<br>◯ Expired<br>◯ Left against medical advice<br>◯ Transfer out |
| Special Care (monitored bed) | ◯ Yes<br>◯ No |
| Days in special bed? | __________________________<br>((in days)) |
| ICU/CCU | ◯ Yes<br>◯ No |
| Days in ICU/CCU | __________________________<br>((in days)) |
| Ward | ◯ Yes<br>◯ No |
| Days in ward | __________________________<br>((in days)) |
| Date of registration in ER | __________________________ |
| Time of registration in ER.<br>(24 hours) | __________________________ |
| Date of disposition from ER?<br><br>(if 'admitted', Source: ED assessment transfer out form) | __________________________ |
| Time of disposition in the ER?<br>(24 hours) | __________________________ |
| Date of hospital discharge<br>(Source: Discharge summary) | __________________________ |
| Time of hospital discharge<br>(24 hours) | __________________________ |
| Did the patient expire in the hospital during stay? | ◯ Yes<br>◯ No |

| | |
|---|---|
| Where did the patient expire? | ◯ ER<br>◯ Special Care<br>◯ ICU/CCU<br>◯ Ward |
| SBP at the time of disposition from the ER? | ____________________<br>((in mm/Hg)) |
| DBP at the time of disposition from the ER? | ____________________<br>((in mm/Hg)) |
| Were discharge instructions related to hypertension provided when the patient was discharged from the hospital? | ◯ Yes<br>◯ No<br>◯ Not documented |
| Did the discharge instructions include any advice on life style modifications (e.g. low salt diet, exercise, etc.) ? | ◯ Yes<br>◯ No |
| Low salt diet? | ◯ Yes<br>◯ No |
| Exercise? | ◯ Yes<br>◯ No |
| Quit smoking? | ◯ Yes<br>◯ No |
| Did discharge instructions include B.P. monitoring advice? | ◯ Yes<br>◯ No |
| Were anti-hypertensive medications prescribed on discharge from ER/ Hospital? | ◯ Yes<br>◯ No<br>◯ Not documented |
| Please name the anti-hypertensive medications and their respective dosages. | ______________________________ |
| Did discharge instructions include follow-up visit? | ◯ Yes<br>◯ No |
| In which service specialty was the follow-up visit of the patient scheduled and when? | ☐ Cardiology<br>☐ Internal medicine<br>☐ Family medicine<br>☐ ED Fast track<br>☐ Neurology<br>☐ Ophthalmology<br>☐ Others |
| Cardiology follow up days | ____________________ |
| Did patient come for the cardiology follow up visit? | ◯ Yes<br>◯ No |

| | |
|---|---|
| What was the follow-up visit date for cardiology? | __________________________________ |
| Internal Medicine follow up days | __________________________________ |
| Did patient come for the Internal Medicine follow up visit? | ◯ Yes<br>◯ No |
| What was the follow up visit date for Internal medicine? | __________________________________ |
| Family Medicine follow up days | __________________________________ |
| Did patient come for the family medicine follow up visit? | ◯ Yes<br>◯ No |
| What was the follow up visit date for Family medicine? | __________________________________ |
| ED Fast Track follow up days | __________________________________ |
| Did patient come for the ED Fast Track follow up visit? | ◯ Yes<br>◯ No |
| What was the follow up visit date for ED Fast Track? | __________________________________ |
| Neurology follow up days | __________________________________ |
| Did patient come for the neurology follow up visit? | ◯ Yes<br>◯ No |
| What was the follow up visit date for Neurology? | __________________________________ |
| Ophthalmology | __________________________________ |
| Did patient come for the ophthalmology follow up visit? | ◯ Yes<br>◯ No |
| What was the follow up visit date for Ophthalmology? | __________________________________ |
| Others (please specify) | __________________________________ |
| Others follow up days | __________________________________ |

| | |
|---|---|
| Did patient come for the "Others" speciality follow up visit? | ◯ Yes<br>◯ No |
| What was the follow up visit date for "Others" speciality? | __________________________ |

# Follow-up questionnaire

| | |
|---|---|
| Date of Discharge | __________________________________ |
| Date of follow up | __________________________________ |
| Date of contact established? | __________________________________ |
| Time of contact established? | __________________________________ |
| Follow up event | ◯ 1st month follow up<br>◯ 3rd month follow up<br>◯ 6th month follow up |
| Contact established?<br>(call made) | ◯ Yes<br>◯ No |
| How many attempts were made to establish contact (through telephonic call)? | ◯ 1st attempt ◯ 2nd attempt<br>◯ 3rd attempt ◯ 4th attempt<br>◯ 5th attempt ◯ 6th attempt<br>◯ 7th attempt ◯ 8th attempt<br>◯ 9th attempt ◯ 10th attempt<br>◯ 11th attempt ◯ 12th attempt<br>◯ 13th attempt ◯ 14th attempt |
| Others, please specify | __________________________________ |
| What were the reasons for no response? | ◯ Phone engaged<br>◯ Phone switched off<br>◯ Not answering<br>◯ Invalid number<br>◯ Number exchanged<br>◯ Wrong number<br>◯ Others |
| Others, Please specify | __________________________________ |
| How are you (is the patient) after getting discharged from the hospital? | ◯ Alive<br>◯ Dead |
| When did the patient expire? | __________________________________ |
| What was the reason for death? | __________________________________ |
| Where did the patient die? | ◯ In hospital<br>◯ At home<br>◯ Others |

| | |
|---|---|
| Others (please specify). | __________________________________ |
| Do you (the patient) regularly monitor your blood pressure readings? | ◯ Yes<br>◯ No |
| How often do you get your blood pressure checked? | ◯ Daily<br>◯ Twice a week<br>◯ Once a week<br>◯ Once a month<br>◯ Randomly<br>◯ On convenience basis (when someone available or BP apparatus is available)<br>◯ Rarely |
| Have you (the patient) tried reducing salt intake since diagnosed with high blood pressure? | ◯ Yes<br>◯ No<br>◯ Unchanged (already using low salt in diet)<br>◯ Others |
| Others, please specify | __________________________________ |
| Do you (the patient) do any physical activity (other than household chores) for at least 30 minutes a day? (E.g. walking, running, jogging, cycling, swimming, gym, etc.) | ◯ Yes<br>◯ No |
| How often do you (the patient) do any physical activity? | ◯ One day a week<br>◯ Two days a week<br>◯ Three days a week<br>◯ Four days a week<br>◯ Five days a week<br>◯ Six days a week<br>◯ Seven days a week (daily) |
| What other exercise or physical therapy do you (the patient) do?<br>E.g. yoga, stretching<br>(Please specify) | __________________________________ |
| Since diagnosis with high blood pressure, did you (the patient) smoke cigarettes? | ◯ Yes<br>◯ No<br>◯ Never smoked |
| How often do you (the patient) smoke cigarettes? | ◯ Daily<br>◯ Not daily<br>◯ Occasionally |
| Were you (the patient) prescribed any blood pressure medications after discharge from the hospital? | ◯ Yes<br>◯ No |
| Are you (the patient) taking those medications regularly? | ◯ Yes<br>◯ No<br>◯ Never |
| Did you (the patient) visit or follow up with your doctor related to blood pressure? | ◯ Yes<br>◯ No |

| | |
|---|---|
| Please specify how often it was? | ◯ Visited once after discharge<br>◯ Visited twice after discharge<br>◯ Visited more than twice after discharge (around once a week) |
| Have you (the patient) developed or suffered from any other illness because of hypertension? | ◯ Yes<br>◯ No |
| What was the illness? | ☐ Stroke<br>☐ Heart attack<br>☐ Kidney disease<br>☐ Eye disease<br>☐ Other illness or condition |
| Other illness or condition<br>(please specify) | __________________________________ |
| Were you (the patient) admitted again since your discharge from the Aga Khan University? | ◯ Yes<br>◯ No |
| What was the reason for your admission to the hospital? | ☐ High blood pressure<br>☐ Chest pain<br>☐ Heart attack<br>☐ Stroke, one sided weakness<br>☐ Headache<br>☐ Kidney failure<br>☐ Others |
| Others<br>(Please specify) | __________________________________ |
